# Designing the content of religious education learning in creating sustainability among children with learning disabilities: A fuzzy delphi analysis

**DOI:** 10.3389/fpsyg.2022.1036806

**Published:** 2022-11-22

**Authors:** Hafizhah Zulkifli, Syar Meeze Mohd Rashid, Suziyani Mohamed, Hasnah Toran, Norakyairee Mohd Raus, Mohd Izzuddin Mohd Pisol, Muhamad Nasri Suratman

**Affiliations:** ^1^Faculty of Education, Universiti Kebangsaan Malaysia, Bangi, Selangor, Malaysia; ^2^Faculty of Quranic and Sunnah Studies, Universiti Sains Islam Malaysia, Nilai, Negeri Sembilan, Malaysia; ^3^Foundation of Quranic Education for Special Children, Nilai, Malaysia; ^4^Faculty of Education, International Islamic University College Selangor, Kajang, Malaysia

**Keywords:** children with disabilities, learning, fuzzy delphi, religious education, Quran, teachers

## Abstract

Teachers found it is hard to figure out what are the best approach and strategies shall be employed to create an effective learning activity that can benefit the children. Children with learning disabilities have distinctive learning difficulties, depending on each individual. Therefore, this requires modification and adaptation in the learning activities to make sure they can learn effectively. Teachers need to make adjustment to the instructions, learning materials, assessments, and activities to accommodate the children with learning disabilities. Therefore, the objective of this research is to develop the content of religious education for children with learning disabilities using fuzzy delphi. This research used method of design and developmental research approach which have three phases. In this research, the researchers focus on the second phase of fuzzy delphi. There were 20 panel experts involved in this research to rank the elements in developing religious education model. Findings showed that, all the elements such as learning style, rights of people with disabilities manners and universal design were above 70% that considered suitable and applicable. It is hoped that this model can assist and guide teachers in teaching religious education for children with learning disabilities.

## Introduction

Religious education teaches children about the ultimate meaning and purpose of life, as well as perspectives on God, moral concerns, faith, self, and reality. In religious education, there are two concepts, namely the general concept and the specific concept of religion (Training and Development Agency for Schools, 2009). Celebration, melancholy, goodness, and forgiveness are the qualities of general concept associated with human experiences. Meanwhile, specific concept such as prayer, worship, and symbol are tied to major faith in all religions. However, teaching and learning religious education is not an easy feat for teachers and children because religious education deals with beliefs and opinion (Buchta et al., 2021). This is hard to digest because it involves abstract concept. Abstract concept refers to the understanding of spirituality, beliefs, opinion, friendship, justice, cooperation, conflict, and empathy. The level of difficulty to teach and learn is doubled when it involves children with learning disabilities (Kpobi and Swartz, 2019; Parr and Stevens, 2019).

Religion shall be taught to typical children as well as children with learning disabilities. Religious education is necessary for children with learning disabilities to participate in spiritual activities to give them the sense of strength and wellbeing (Hakiman et al., 2021). Furthermore, it benefits children with learning disabilities by assisting them to develop positive attitude toward others, respecting their ideas and experiences, reflecting on and considering their own and others’ values, and making sense of their environment where they live as individuals and members of groups (Training and Development Agency for Schools, 2009). Religious education also encourages children with learning disabilities to engage in religious beliefs and practices (Poston and Turnbull, 2004; Brooke and Smith, 2009). Besides, it serves as a clinical intervention medium for children with learning disabilities (Schaap-Jonker et al., 2013). There is a huge expectation that religious education provided to children with learning disabilities would leave a good impact on them.

A study revealed the lack of reference to religious education for children with learning disabilities becomes an obstacle for both teachers and children (Hanum, 2014). Teachers found it is hard to figure out what are the best approach and strategies shall be employed to create an effective learning activity that can benefit the children. This situation indirectly put the children at a disadvantage because they could not gain understanding and knowledge with the teaching approaches and strategies that do not fit to the level that they can learn. Children with learning disabilities have distinctive learning difficulties, depending on each individual. Therefore, this requires modification and adaptation in the learning activities to make sure they can learn effectively. Teachers need to make adjustment to the instructions, learning materials, assessments, and activities to accommodate the children with learning disabilities so that the curriculum requirements are met and the learning process is optimised (Onwumere et al., 2021).

Teachers shall consider a wide range of opportunities for children with learning disabilities to exhibit what they know and can do, because they learn and understand in different ways than their typical counterparts. Furthermore, teachers must identify strategies to minimise or eliminate barriers so that all children with learning disabilities are not left behind to participate and learn (Wilson and Landa, 2019). Teachers must concentrate on how children with learning disabilities learn because they have different ways of learning and acquiring knowledge. Positive reinforcement or praise is a simple yet effective strategy to spark children’s interest in learning more about religion (Aung, 2020; Hanafi et al., 2020). Furthermore, an engaging learning resource that is easy to access and neatly labelled encourages autonomous use. This may help them have a better understanding of religious beliefs, practices, and experiences.

Children with learning disabilities benefit most from multisensory strategies. Role playing, pretend play, simulations, field excursions to religious sites, and sharing special meals that represent the religion are all useful for the children (Ozcan and Merdan, 2020; Wright et al., 2020). These activities can facilitate children with learning disabilities to comprehend abstract concepts such as faith in God. Furthermore, showing high-quality artefacts enables children with learning disabilities to understand the features of a variety of faiths. Artefacts are useful to assist children to gain a better understanding of religious rituals and sacred objects.

Also, visual learning materials such as digital presentations, videos, and graphics, can help children with learning disabilities pay attention to the learning session. The use of video and digital presentations of situations involving moral dilemmas is a powerful aid to increase children’s engagement in the classroom effectively (Ozcan and Merdan, 2020; Wright et al., 2020) and create a responsive learning environment (Training and Development Agency for Schools, 2009). To make the learning session easier to understand for students, teachers can demonstrate the steps of worship such as ablution and prayer using colourful and big-size graphic cards. Not only that, but a photo of a holy place also hung on the classroom wall can help children with learning disabilities to recall and think about religion. Besides, virtual reality graphics are useful as an alternative learning tool to increase children’s engagement (Cihak et al., 2016).

Collaboration among special education and religion teachers as well as parents is crucial in determining the content of learning activities as well as teaching techniques and strategies (Ekinci and Acar, 2019; Majoko, 2019). Learning activities are planned based on assessments, observations, and discussions with the team. Teachers use the findings to organise, deliver, and assess the outcome of worship learning activities. The learning outcomes are determined according to the needs of the students rather than the needs of the teachers.

In Malaysia, religious education is a compulsory subject at all education levels, from early childhood to the upper secondary school. Religious education is covered in two subjects, Islamic Education and Moral Education under the National Preschool Curriculum Standard, Primary School Standard Curriculum, and Secondary School Standard Curriculum (Ministry of Education Malaysia, 2014). Moral Education is taught to non-muslim children while Islamic Education is taught to the Muslim children. Furthermore, religious education is incorporated in the special education curriculum such as the National Preschool Curriculum Standard (Special Education), as well as the Primary and Secondary School Standard Curriculums (Special Education).

This study aims to identify the threshold values and fuzzy evaluation.

## Literature review

Education for all can be defined as the right of all students to be provided with education regardless of their social background or intellectual development. Therefore, curriculum adaptation does occur in the context of special education in Malaysia. The need to provide education to all students is one of the utmost social responsibilities of a society. This adaptation enables students with learning disabilities consisting of various spectrum of Down syndrome, developmental delay, autism, and hyperactivity as well as other types of disabilities to enroll in the Integrated Special Education (Learning Disabilities) programmed in Malaysia (Ahmad, 2018).

The Special Education Curriculum is developed so that the lesson gained in the classroom can be applied in daily life. In addition, the special education curriculum provides an optimal quality education plan for students with learning disabilities so that they can function as independent individuals, succeed in life, and contribute to society. In Malaysia, the curriculum for Special Education for Students with Learning Disabilities is manifested in two documents, namely the Primary School Standard Curriculum and the Description of the Integrated Special Education Syllabus for Students with Learning Difficulties at both Primary and Secondary Schools. There are four main areas that need to be imparted which are life management, functional academics, social, leisure and creativity as well as spirituality and values. In the field of spirituality and moral values, there is an Islamic Education (Religious Education) component that focuses on imparting knowledge, skills, and appreciation of Islam according to the Quran and the Sunnah.

In teaching religious education to students with learning difficulties, it is vital to adopt several models and theories that can help this group to acquire a meaningful education. Among the theories that can be applied is the cognitive learning theory by Vygotsky, known as Vygotsky’s Theory of Cognitive Development from the sociocultural perspective, was born in Russia and holds a Law degree from Moscow University. His theory applies the scaffolding concept via the zone of proximal development. This zone of proximal development distinguishes a student’s actual development from the anticipated potential development. This zone of proximal development refers to functionality that is not yet mature but is undergoing a maturation process (Vygotsky, 1978). When children interact with individuals who are more knowledgeable, it means that they are working in their zone of proximal development, which is a situation where an individual is not able to perform a task on his/her own but can make it work with the help of others (Ahmad, 2018).

This strategic planning and collaboration involves parents and peers. This is because social interaction and language influence the thinking of a learner as a result of social and cultural experiences. Social interaction is important for cognitive development and Vygotsky argued that children shall acquire knowledge and concepts from individuals who are more knowledgeable than them.

This theory helps students with learning disabilities to learn new skills because in to get through the zone of proximal development, they need to be facilitated by their teacher via learning activities that have been deliberately planned according to the student’s ability level. For example, in the Islamic Education subject, students are required to memorise to learn, therefore they shall be guided by their teacher to memorise the prayer, either by using teaching aids such as flash cards or others. Students can learn new skills gradually such as memorisation and teachers shall provide support in memorising activities using proper teaching aids.

Furthermore, to teach special needs students with learning difficulties, behavioural learning theory is applied. This theory emphasises the actual change in behaviour due to the association between stimulus and response. The main construct of this theory is the response to reinforcement provided during learning, this idea was pioneered by Thorndike, Watson, and Skinner.

## Methodology

The researchers conducted this study using the Fuzzy Delphi method. Murray et al. (1985) introduced this method, which combines the Fuzzy numbering set and the Delphi method. This means that the Fuzzy Delphi method is an improvised version of the Delphi method. The improvisation somehow improve the technique’s effectiveness as a measuring tool, as it is deemed capable of solving problems with precision and unpredictability in a study.

Furthermore, the Delphi Fuzzy method is synthesized from the traditional Delphi and fuzzy set theory. This fuzzy set theory was introduced by Zadeh (1965), a mathematician and researcher in 1965. In addition, this theory is an extension of the classical set theory, in which each element of a set is evaluated using a binary set (Yes or No). Not only that, but the fuzzy set theory also enables gradual evaluation of each element in a set of values contained within a fuzzy set, i.e., from 0 to 1 or by unit interval (0, 1). The Fuzzy Delphi technique is capable of processing ambiguity of the predictive items, the respondents’ information, and the participants’ individual characteristics. In a nutshell, the Fuzzy Delphi method is used to ascertain consensus among experts who serve as respondents by applying quantitative methods.

### Sampling

Purposive sampling was used to select the relevant experts. The panel consists of highly qualified experts in Children with learning disabilities and special education. The panel of experts is made up of 20 experts who agreed to take part in the study. If the experts chosen are less proficient about the study subjects, the credibility of the study is jeopardized. As a result, several selection criteria are determined in the selection panel as follows:

(1) Have a minimum of 5 years of experience in teaching children with learning disabilities and religion.

(2) Have skilled in teaching in special education for religion.

(3) Actively involved as trainer for special education in religious education.

### Participants

The 20 experts consist of experts from Quranic Field, Islamic Education Field, non-government organization and policy makers are selected to answer the questionnaire. Experts in fuzzy method in is non-probability sampling or judgement sampling (Mustapha and Darussalam, 2018) based on their expertise. The details of the expert are as follows in Table 1.

**TABLE 1:** List of the participants.

No.	Participant	Expert field	Place of work
1	A001	Quran field	Universiti Sains Islam Malaysia
2	A002	Quran field	Universiti Kebangsaan Malaysia
3	A003	Quran field	International Islamic University College Selangor
4	A004	Quran field	Universiti Pendidikan Sultan Idris
5	A005	Quran field	International Islamic University Malaysia
6	A006	Islamic education field	Universiti Kebangsaan Malaysia
7	A007	Islamic education field	Institute of Teacher Education
8	A008	Islamic education field	Universiti Teknologi Mara
9	A009	Islamic education field	Universiti Teknologi Malaysia
10	A010	Islamic education field	International Islamic University Malaysia
11	A011	Non-government organization	Malaysia Federation of Deaf
12	A012	Non-government organization	Foundation of Quranic for special needs
13	A013	Non-government organization	Celebral Palsy Malaysia
14	A014	Special education field	Institute of Teacher Education
15	A015	Special education field	International Islamic University Malaysia
16	A016	Special education field	Universiti Kebangsaan Malaysia
17	A017	Special education field	
18	A018	Policy makers	Special Education Department, Ministry of Education
19	A019	Policy makers	Special Education Department, Ministry of Education
20	A020	Policy makers	Special Education Department, Ministry of Education

### Instrument

This instrument consists of five elements on the content of religious education namely (1) Rights to learn for Children with learning disabilities, (2) Access to support learning religious education, (3) Universal design learning for Children with learning disabilities, (4) Learning style for children with learning disabilities and, (5) attitude in learning for Children with learning disabilities. This research used fuzzy Delphi Questionnaire with 38 items with seven points fuzzy linguistic scale; strongly disagree, disagree, somewhat disagree, neither agree or disagree, somewhat agree, agree, and strongly agree A reliability test was conducted for this study and its Cronbach’s alpha for internal reliability was 0.73.

The experts were given a set of questionnaires and they need to fulfill the answer according to the seven point of agreement. Once they had done the answer, the experts returned the instrument to the researcher.

### Procedure of fuzzy delphi methods

The fuzzy delphi methods steps are as follows:

#### Step one: Determination of experts or number of experts involved

In total, 20 experts were selected to answer the questionnaire instrument.

#### Step two: Linguistic scale selection

**FIGURE 1:**
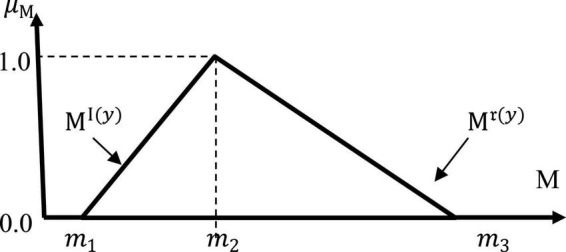
Fuzzy triangle number.

This process involves the process of converting all linguistic variables into fuzzy triangular numbering. This step involves the conversion of linguistic variables to the addition of fuzzy numbers. The numbering of fuzzy triangles represents the value *m_1_*,*m_2_*, dan *m_3_* and written like this (*m_1_*, *m_2_*, and *m*_3_). Value *m_1_* represents the minimum value, value *m_2_* represents reasonable value, while value *m_3_* represents the maximum value. Meanwhile, fuzzy triangle numbering is used to produce a fuzzy scale for the purpose of translating linguistic variables into fuzzy numbers. The number of levels for the fuzzy scale is in odd numbers. This can be explained in Figure 1.

There are two types of Linguistic point scales, namely the five-point linguistic scale and the seven-point scale. Therefore, in this study, the researcher chose a seven-point linguistic scale consisting of strongly disagree, disagree, more or less disagree, undecided, more or less agree, agree, and strongly agree. The Table 2 shows the seven-point linguistic scale used.

**TABLE 2:** Seven points of linguistic scale and fuzzy scale.

Number of Likert scale	Interpretation of Likert scale	Fuzzy scale		
		m_1_	m_2_	m_3_
1	Strongly disagree	0.00	0.00	0.10
2	Disagree	0.00	0.10	0.30
3	More or less disagree	0.00	0.30	0.50
4	Undecided	0.03	0.50	0.70
5	More or less agree	0.50	0.70	0.90
6	Agree	0.70	0.90	1.00
7	Strongly agree	0.90	1.00	1.00

The basic to transform the seven-point linguistics scale into the specifics fuzzy numbers is that, the fuzzy scale is not at a fixed value as in the Likert scale. This shows that if using the Likert scale each choice by the expert or the respondent only chooses a whole number, and it is fixed and the interpretation is one-way only. However, if the expert chooses a value of 1 in the Likert scale then it shows the answer and data from the respondent only strongly disagree. For the fuzzy scale if an expert or respondent chooses a value in the Likert scale in the questionnaire, when the data is translated into a fuzzy scale, the translation of the chosen scale will be categorized into three values which consists of minimum value, reasonable value and maximum value. This shows that the translation of the data in the fuzzy scale is not at one value. This is what differentiates the use of a fuzzy scale compared to a Likert scale (Mohd Jamil et al., 2017). Table 3 shows the difference interpretation of Likert scale and fuzzy scale.

**TABLE 3:** Different interpretation of Likert scale and fuzzy scale.

Expert choice	Likert scale	Interpretation of Likert scale	Fuzzy scale	Interpretation of fuzzy scale
Seven point Likert scale	7	Strongly agree	0.9 Minimum value (m_1_)	The agreement value is likely to be on a scale of 0.9 or 90% agree
			1.0 Reasonable value (m_2_)	The agreement value is likely to be on a scale of 1.0 or 100% agree
			1.0 Maximum value (m3)	The agreement value is likely to be on a scale of 1.0 or 100% agree

#### Step three: Get the average value or threshold value (d)

After the researcher successfully obtained all the data and information from the experts, the researcher converted all Likert scales to fuzzy scales. All data and information were analyzed using Microsoft Excel software. There is three conditions can be used to accept all the item agreed by the experts whether the item is discarded or accepted based on expert agreement. According to Mohd Jamil et al. (2017), researchers can use these three conditions, which are condition 1: Using a threshold value, condition 2: Based on the traditional delphi method and condition 3: Based on α-cut value. The formula for condition 1 using threshold value is:


$$d\,\left(\overset{˘}{m},\overset{˘}{n}\right)=\sqrt{\frac{1}{3}}\,\left[{\left(m_{1}-n_{1}\right)}^{2}+{\left(m_{2}-n_{2}\right)}^{2}+{\left(m_{3}-n_{3}\right)}^{2}\right]$$

To get the total threshold value or threshold value (*d*) is by reading like this *m_1_* is the minimum average value for each expert minus with *n_1_*, which is the minimum element value of each expert, then squared. Then, add with *m_2_* is a reasonable average value for each expert minus with *n_2_* that is, the reasonable element value of each expert, then squared. After that, add with *m_3_* is the maximum average value for each expert minus with *n_3_* which is the maximum element value of each expert, then squared. The result of the average value and the element value of the minimum, reasonable and maximum is multiplied by one third and the root of the power. The threshold value is *d* ≤ 0.2, means that the item accepted. If the threshold value is *d* > 0.2 which is more than 0.2 the item will be rejected or a second round only against experts who disagree (Mohd Jamil et al., 2017).

Next, for condition 2: Based on traditional delphi method state that, if expert group consensus accepted items more than 75% means that item were accepted either used or discarded the item. On the other hand, if the consensus of the experts taken by the researcher reaches less than 75%, then, the researcher has to do a second round to ensure that the consensus group is agreed (Mohd Jamil et al., 2017).

#### Step four: Defuzzification process

Defuzzification Process is to determine the ranking of the item using condition 3 which is Based on α-cut value. Means that, α-cut is an average number between fuzzy number around 0–1, so the α-cut value is 0.5 (Tang and Wu, 2010; Mohd Jamil et al., 2017; Mustapha and Darussalam, 2018). If the A_max_ fuzzy score value is equal or more than 0.5, then the measured item is accepted, and if less than 0.5, then, the measured item is rejected. The symbol of deffuzification process is A_max_. There were three formula can be used in defuzzification process and researcher can choose either one. The formula was the following:


$${\text{A}}_{\text{max}}=1/3\times \left({\text{m}}_{1}+{\text{m}}_{2}+{\text{m}}_{3}\right)$$

$${\text{A}}_{\text{max}}=1/4\times \left({\text{m}}_{1}+{\text{m}}_{2}+{\text{m}}_{3}\right)$$

$${\text{A}}_{\text{max}}=1/6\times \left({\text{m}}_{1}+{\text{m}}_{2}+{\text{m}}_{3}\right)$$

### Analysis of the data

**FIGURE 2:**
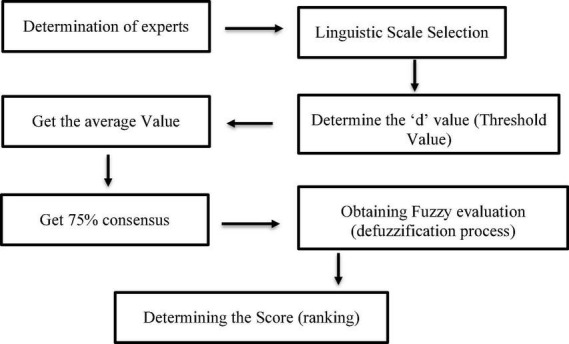
Schematic work flow diagram of the consensus development process.

In this research, researcher used Microsoft Excell template to analyze the fuzzy Delphi data. The researcher has followed the course of fuzzy Delphi analysis and bought the analysis book (Mohd Jamil and Mat Noh, 2021). Then, the researcher uses a QR code to access the Excel template complete with fuzzy formulas. The researcher only enters the data and analyze the result of the fuzzy number. This template is like an SPSS template where the researcher only enters the data and analyzes the data. All formulas have been placed in SPSS. The researcher, key in the raw data into the excel according seven point of fuzzy scale. The responses were transformed from linguistic variables to fuzzy scales. Then the researcher see threshold values of ≤0.2. It means that the item achieved good consensus if the threshold values below 0.2 and the consensus must be at least 75% (Mustapha and Darussalam, 2018). Then, defuzzification is used to convert these values to real numbers for the purpose of ranking their importance. The defuzzification number must higher than 0.5 to be accepted and if it is below 0.5 the item should be dismissed. The analysis can be illustrate in Figure 2.

## Results and discussion

### Threshold value

To figure out the degree of expert consensus, all items were analyzed by determining the distance between the Fuzzy numbers to determine the threshold value, *d*. During data analysis, the distance between two Fuzzy numbers was calculated by measuring the deviation of the mean values between the experts. The degree of consensus shall have a threshold value <0.2 or at least 75% to obtain the expert consensus value.

Table 4 shows the threshold values and percentage of expert consensus for the content of Religious Education learning for Children with learning disabilities. The findings show that the first condition is already complied with because the threshold value for all items is lesser or equal to 0.2 (≤0.2). Next, the second condition is complied with because the consensus value of the expert group for each item is more than 75%, where all the 38 items are between 90 and 100%. This indicates the degree of consensus between experts has reached an excellent level. Therefore, the second round of Fuzzy Delphi is not required because the data acquisition has met both conditions.

**TABLE 4:** Findings of threshold values of the content religious education learning for children with learning disabilities.

(a) Rights to learn for children with learning disabilities			
Item	Subject	Threshold value	Expert consensus percentage (%)
A1	The obligation of the disabled to study religion is the same as the typical human beings	0.196	90
A2	Being disabled is not a barrier for Children with learning disabilities to learn the religion	0.059	95
A3	Children with learning disabilities can learn the religion according to appropriate methods and techniques	0.037	95

**(b) Access to support to learn religion for children with learning disabilities**			

**No.**	**Subject**	**Threshold value**	**Expert consensus percentage (%)**

B1	Islamic Administrative Institution (Religious Department)	0.027	100
B2	Mosque Institution	0.027	100
B3	Non-governmental organisations	0.027	100
B4	Preacher	0.093	90
B5	Activist	0.068	95
B6	Neighbourhood	0.104	95
B7	Volunteers	0.120	95
B8	Parents	0.157	95
B9	Family	0.120	95
B10	Education Institution	0.120	95

**(c) Universal design learning for children with learning disabilities**			

**No.**	**Subject**	**Threshold value**	**Expert consensus percentage (%)**

C1	The infrastructure provided to learn the religion is in accordance with the needs of the disabled	0.247	80
C2	The teaching materials used in the learning of religion are in accordance with the needs of the disabled	0.215	85
C3	The learning method of the religion is suitable for the needs of the disabled	0.193	85
C4	Religion learning curriculum is suitable for the needs of the disabled	0.164	95
C5	The form of support to learn the religion is in accordance with the needs of the disabled	0.190	85
C6	The communication used to learn the religion is in line with the needs of the disabled	0.172	90
C7	The assessment conducted is in accordance with the strengths of the disabled	0.202	90
C8	The infrastructure provided can be used by all categories of disabled people	0.223	85
C9	Teachers can adapt the curriculum according to the needs of the disabled	0.137	95

**(d) Learning style for children with learning disabilities**			

**No**	**Subject**	**Threshold value**	**Expert consensus percentage (%)**

D1	Children with learning disabilities can learn religion visually according to their ability	0.027	100
D2	Children with learning disabilities can learn religion visually according to their ability	0.027	100
D3	Children with learning disabilities can learn religion by reading and writing depending on their ability	0.027	100
D4	Children with learning disabilities can engage in kinesthetic learning of the religion according to their ability	0.027	100
D5	Children with learning disabilities can learn religion by combining more than one learning style depending to their ability	0.027	100

**(e) Attitude learning for children with learning disabilities**			

**No**	**Subject**	**Threshold value**	**Expert consensus percentage (%)**

E1	Ensure the person, clothing, and place is in a neat and clean condition	0.059	95
E2	Selection of appropriate time	0.085	95
E3	Covering the *aurat* while studying religion	0.118	90
E4	It is fun to study religion	0.118	90
E5	Respect and glorify the *mushaf*	0.090	95
E6	Praying to avoid accidents	0.115	95
E7	Praying before learning	0.110	90
E8	Read the scriptures with civility	0.174	95
E9	Read the scriptures	0.161	90
E10	Trying to read and memorise scriptures	0.118	90
E11	Respect teachers who teach religion	0.083	95

### Fuzzy evaluation of the content of religious education learning for children with learning disabilities

The analysis shows the learning content designed for Children with learning disabilities to learn religion. In this questionnaire, there are 38 items. There were five construct in this questionnaire.

According to Table 5, all items have a high expert consensus of more than 0.600. This means that all 38 component items of religious education for children with learning disabilities have been agreed upon by all experts. Item A3 (defuzzification: 0.953) rank as number one by the experts while item A1 (defuzzification: 0.873) ranks as number three and A2 (defuzzification: 0.943) rank as number two by the experts.

**TABLE 5:** Fuzzy evaluation on construct one: The rights to learn for children with learning disabilities.

		Fuzzy evaluation				
Item	Design of model content	m_1_	m_2_	m_3_	Defuzzification	Position
A3	Children with learning disabilities can learn the religion according to appropriate methods and techniques	0.880	0.985	0.995	0.953	1
A2	Being disabled is not a barrier for Children with learning disabilities to learn the religion	0.860	0.975	0.995	0.943	2
A1	The obligation of the disabled to study religion is the same as the typical human beings	0.770	0.900	0.950	0.873	3

Table 6 shows that the rank for the construct two: access to support to learn religion for Children with learning disabilities firstly from B10 refer to education institutions (defuzzification: 0.930), secondly, followed by parents (defuzzification: 0.928), thirdly by volunteers (defuzzification: 0.903) and fourthly by Islamic administrative institution (defuzzification: 0.897). Next, non-government organization and activist share the same rank which is rank 5 (defuzzification: 0.895) followed by family rank 6 (defuzzification: 0.892), preacher and mosque institution share the same rank which is rank 7 (defuzzification: 0.850) and neighborhood rank 8.

**TABLE 6:** Fuzzy evaluation on construct two: Access to support to learn religion for children with learning disabilities.

		Fuzzy evaluation				
Item	Design of model content	m_1_	m_2_	m_3_	Defuzzification	Position
B10	Education Institution	0.840	0.960	0.990	0.930	1
B8	Parents	0.840	0.960	0.985	0.928	2
B7	Volunteers	0.790	0.935	0.985	0.903	3
B1	Islamic Administrative Institution (Religious department)	0.810	0.925	0.955	0.897	4
B3	Non-governmental organization	0.770	0.925	0.990	0.895	5
B5	Activist	0.770	0.925	0.990	0.895	5
B9	Family	0.790	0.920	0.965	0.892	6
B4	Preacher	0.730	0.875	0.945	0.850	7
B2	Mosque Institution	0.740	0.875	0.935	0.850	7
B6	Neighborhood	0.670	0.830	0.920	0.807	8

The findings of Table 7 show that all experts have agreed on the entire item for access to universal design facilities for learning. Based on the findings, item C9 has the highest position with defuzzification value of 0.897. This demonstrates that the suitability of the learning curriculum is significant and vital to meet the needs of students with disabilities. In addition, experts have found that apart from curriculum adaptation, learning methods (C3: 0.863), forms of learning support (C5: 0.858), teaching materials (C2: 0.853), assessment (C7: 0.845), infrastructure (C7: 0845) and infrastructure (C1: 0.830) shall be provided and relevant to the needs of students with disabilities. This is important so that the learning of religious education for students with disabilities can be provided well, in a comfortable and conducive manner.

**TABLE 7:** Fuzzy evaluation on construct three: Universal design learning for children with learning disabilities.

		Fuzzy evaluation				
Item	Design of model content	m_1_	m_2_	m_3_	Defuzzification	Position
C9	Teachers can adapt the curriculum according to the needs of the disabled	0.790	0.925	0.975	0.897	1
C6	The communication used to learn the religion is in line with the needs of the disabled	0.760	0.900	0.960	0.873	2
C4	Religious learning curriculum is suitable for the needs of the disabled	0.750	0.895	0.965	0.870	3
C3	The learning method of the religion is suitable for the needs of the disabled	0.750	0.890	0.950	0.863	4
C5	The form of support to learn the religion is in accordance with the needs of the disabled	0.740	0.885	0.950	0.858	5
C2	The teaching materials used in the learning of religion are in accordance with the needs of the disabled	0.740	0.880	0.940	0.853	6
C7	The assessment conducted is in accordance with the strengths of the disabled	0.720	0.870	0.945	0.845	7
C8	The infrastructure provided can be used by all categories of disabled people	0.710	0.860	0.935	0.835	8
C1	The infrastructure provided to learn the religion is in accordance with the needs of the disabled	0.710	0.855	0.925	0.830	9

Table 8 shows construct learning style for Children with learning disabilities. The first rank is D5 (defuzzification: 0.918), second rank followed by D4 (defuzzification: 0.908), third rank was D1 (defuzzification: 0.880). Next, D3 (defuzzification: 0.872) at rank 4 and D2 (defuzzification: 0.870) at rank 5.

**TABLE 8:** Fuzzy evaluation of construct four: Learning style for children with learning disabilities.

		Fuzzy evaluation				
Item	Design of model content	m_1_	m_2_	m_3_	Defuzzification	Position
D5	Children with learning disabilities can learn religion by combining more than one learning style according to their ability	0.810	0.950	0.995	0.918	1
D4	Children with learning disabilities can engage in kinesthetic learning of the religion according to their ability	0.800	0.940	0.985	0.908	2
D1	Children with learning disabilities can engage in visual learning of the religion according to their ability	0.760	0.910	0.970	0.880	3
D3	Children with learning disabilities can learn religion by reading and writing according to their ability	0.750	0.900	0.965	0.872	4
D2	Children with learning disabilities can engage in auditory learning of the religion according to their ability	0.750	0.900	0.960	0.870	5

Based on the findings Table 9, all experts strongly agree with all the items. They agreed that to impart a great deal of learning values or attitude to students with disabilities, cleanliness should be the priority. This is reflected when item E1 gets the highest consensus with a defuzzification value of 0.947. However, all experts strongly agree with all the items, which show the various practices of good values shall be instilled into students with disabilities. Among them are the value of respecting teachers (E11: defuzzification 0.933), glorifying the scriptures (E5: defuzzification 0.928), keeping time (E5: defuzzification 0.923), praying before studying (E7: defuzzification 0.920), praying to avoid accidents (E6: 0.918), covering the *aurat* (E3: defuzzification 0.910), making learning fun (E4: defuzzification 0.910), trying to read and memorize the scriptures (E10: defuzzification 0.910), read the scriptures (E9: defuzzification 0.985), and read the scriptures with civility (E8: defuzzification 0.888).

**TABLE 9:** Fuzzy evaluation on the construct five: Attitude learning for children with learning disabilities.

		Fuzzy evaluation				
Item	Design of model content	m_1_	m_2_	m_3_	Defuzzification	Position
E1	Ensure the person, clothing, and place is in a neat and clean condition	0.860	0.975	0.995	0.943	1
E11	Respect the teachers who teach religion	0.850	0.965	0.985	0.933	2
E5	Respect and glorify the *mushaf*	0.840	0.960	0.985	0.928	3
E2	Selection of appropriate time	0.820	0.955	0.995	0.923	4
E7	Praying before learning	0.830	0.950	0.980	0.920	5
E6	Praying to avoid accidents	0.830	0.950	0.975	0.918	6
E3	Covering the *aurat* while studying religion	0.810	0.940	0.980	0.910	7
E4	It is fun to study religion	0.810	0.940	0.980	0.910	7
E10	Trying to read and memorise the scriptures	0.810	0.940	0.980	0.910	7
E9	Read the scriptures	0.800	0.925	0.960	0.895	8
E8	Read the scriptures with civility	0.790	0.915	0.960	0.888	9

## Discussion

From the study findings using the fuzzy Delphi technique, it demonstrates the position of the constructs of the Quranic teaching model in the Islamic Education subject for special needs students with learning difficulties, indicating the Access Construct – support and learning style show the highest defuzzification value of 0.957, whereas the Access Construct – universal design has the lowest defuzzification value which is 0.910. This is followed by the Rights of Disabled to Learn Construct at 0.953 and the Manners Construct at 0.943.

Access to support is deemed as the most significant in shaping the Quranic teaching model in the Religious education subject. This support includes support from parents, educational institutions, non-profit organisations, and so on. Also, this kind of auxiliary services is so important to provide the level playing field for students with special needs so that they have the same opportunities as their peers (Koo and Khairuddin, 2021). According to Amin et al. (2020), there are eight forms of auxiliary services received by families with children with learning disabilities. The support services are: (1) Community Rehabilitation Programme, (2) Allowance or Financial Assistance, (3) Early Intervention, (4) Security (Provision of Conducive Infrastructure), (5) Social Support, (6) Bus Service, (7) Counselling and Advisory Services, and (8) Treatment and Medicine. For special needs students with learning difficulties who require lower degree of continuous support in daily living activities so that they can participate in the community in terms of mobility, communication, self-care, work as well as living a quality life as a member of society.

In addition, the learning style is also crucial in shaping the Quranic teaching model in religious education for special needs students with learning difficulties. The students’ learning style depends on their ability level and needs. The strategy used to devise the curriculum according to the level of special needs students with learning difficulties is the example of holistic learning, where the teacher can accommodate, by taking an action in making adaptations or modifications that makes learning more relevant and customised to students with special needs, as stipulated by the law. Modification is adjusting learning so that it can be carried out realistically and in accordance with the individuals. Adaptation can be carried out by adjusting the content, teaching aids, and teaching techniques. For instance, the study by Lee et al. (2021) modified mathematical strategies to fulfil the needs of the students. The mathematical strategies are modified via diverse multiplicative concepts and fitting the form of representations. The lesson plan is designed by setting the expectations to accommodate the needs of the students by managing instructional structure and progress as well as adjusting the cognitive demand of tasks. Same goes to Larco et al. (2021), where they modified learning to suit dyslexia children (one of the categories of learning disabilities) by creating a website and mobile app as the preliminary step toward the diagnosis and treatment of dyslexic children. Such app provides didactic educational games and activities to improve literacy skills for students with reading disability. The app can be a valuable tool for children with dyslexia to progress well at school, increase their self-confidence, thus helping them reach their full potential and able to make a positive contribution to society when they become adults. Moreover, due to the requirements during the COVID-19 pandemic, Mateos-Sanchez et al. (2022) had created a chatbot. Chatbot is a software tool that allows a conversation to be continued in automatically between the user and the machine via mobile application. Chatbot is characterised by its features, it can be an educational ICT tool that introduces innovation, inclusion, and quality to be integrated into education for people with intellectual disabilities to facilitate them in terms of social skills.

The construct of learning rights is the second highest construct according to the fuzzy value. The rights to education is constituted in the Laws of Malaysia - Act 550, which is the Education Act 1996 and the (Special Education) Regulations 2013 which provide that: “Students with special needs require a programme provided either in special schools for visually impaired or hearing impaired students, integrated programmes in regular schools for students with visual impairment or hearing impairment or learning disabilities, and inclusive education programmes for students with special needs who can attend lessons in a regular classroom with regular students”. Therefore, teachers need to modify the activities and the curriculum itself to fulfil the rights of special education children to learn. For example, organising inclusive education and career transition programmes. Setting aside students with learning disabilities from their normal friends seems to deny their rights to education (Ahmad, 2018; Mohd Khalil et al., 2022).

Moreover, manners is the third highest construct according to the fuzzy ranking value. The value of this manners can be categorised into five aspects to be emphasised, namely (i) Manners in life. (ii) Manners in socialising or interacting with people. (iii) Ethics while worshipping. (iv) Ethics in learning. (v) Manners toward Allah SWT and Rasulullah SAW. In this study context, the value of good manners in learning the Quran shall be emphasised. The purpose of moral education is to inculcate humans to have a great interaction by restraining from indecent behaviour. In the situation of disabled people with learning disabilities, moral education helps them to learn to be independent without having excessive shame due to the lack of self-worth. With good manner that are in accordance with local community norms, disabled people with learning disabilities are more well received as members of society who need to be supported, protected, and loved. The emphasis that shall be given in inculcating the manners of disabled with learning disabilities is that a person’s value lies in their religion and manners, not in the shortcomings and disabilities that can incapacitate their spirit. The importance of moral education for this group is to circumvent feelings of despair with what they are lacking in due to lack of knowledge and religious constraint. This also enables disabled individuals with learning disabilities to make judgement about any action taken, whether it is right or wrong as a guide in living a life according to Shariah (Md Yusoff and Awang, 2019).

Next, the Access construct: Universal Design for Learning is the last one in the fuzzy analysis ranking. This is probably because the experts think that special needs children with learning difficulties shall also have a learning design that matches their level of ability. This universal design encompasses the adaptation of curriculum, communication, learning methods, forms of support, teaching materials, assessment, infrastructure for the special needs students with learning difficulties that match their needs in learning Islamic religious education, especially in the field of the Quran. The Universal Design concept is derived from the field of architecture and is driven by the accessibility goal (Story et al., 1998). Universal design in learning (UDL) is suitable for special needs children with learning difficulties, especially those who enrol in inclusive education. This is because UDL recognises the need to create opportunities for the inclusion of diverse learners by providing curricular and instructional activities that facilitates multiple means of representation, expression, and engagement (King-Sears, 2009). In its early years, UDL focused on the use of technology to facilitate accessibility. More recent development of the theory and practice of UDL recognises instructional pedagogies that facilitate accessibility for diverse learners (Burgstahler, 2009). UDL is proven to support access, participation, and progress for all types of learners (Katz, 2013).

The necessity of applying fuzzy delphi is saving times, the agreement rates is better than the old version and the guarantee of obtaining expert agreement is higher. Fuzzy Delphi also to minimize the fuzzy round rate (Mustapha and Darussalam, 2018).

Implication of this study is this study is a first steps to create the content for religious education learning in order to create a model for Children with learning disabilities. It is also as guidance to the teacher to teach and do activities to children with learning disabilities based on the elements provided.

## Limitations

The limitation of this study is that this study only uses the Delphi fuzzy technique to design the Islamic Education teaching model in the field of the Quran. Further studies can employ other approaches such as the nominal group technique or the Interpretive Structural Modelling technique. The respondents involved in this study are experts in the field of education, special education, lecturers, teachers, officers and heads of the Special Education sector. Future studies can involve the disabled themselves and their parents.

## Conclusion

In a nutshell, the development and design of the Islamic Education model in the field of the Quran shall emphasis the importance of access to support, learning style, learning rights of the disabled, manners, and universal design. Therefore, the way of learning shall be adapted to fulfil the wishes and needs of special needs students with learning difficulties according to their functional level, which can be mild, moderate, or severe.

## Data availability statement

The original contributions presented in this study are included in the article/Supplementary material, further inquiries can be directed to the corresponding author.

## Author contributions

SM and NR: introduction. SR and MS: literature review. SR and HZ: methodology. HZ, SR, and HT: findings. HZ, MP, and NR: discussion. MP: implication. HZ: conclusion. All authors contributed to the article and approved the submitted version.

## References

[B1] Ahmad N. A. (2018). Inclusive education: Better and for the best. *Int. J. Acad. Res. Progress. Educ. Dev.* 7 557–568. 10.6007/IJARPED/v7-i3/4574

[B2] Amin A. S. Ayu H. M. Manap J. (2020). Perkhidmatan sokongan bagi keluarga yang mempunyai anak masalah pembelajaran di luar bandar. *Jurnal Psikologi Malaysia* 34 28–41.

[B3] Aung Y. M. (2020). Humanism and education. *Int. Adv. Res. J. Sci. Eng. Technol.* 7 13555–13562.

[B4] Brooke B. A. Smith D. J. (2009). Multiculturalism, religion, and disability: Implications for special education practitioners. *Educ. Train. Autism Dev. Disabil.* 44 295–303.

[B5] Buchta R. Cichosz W. Zellma A. (2021). Religious education in Poland during the COVID-19 pandemic from the perspective of religion teachers of the Silesian Voivodeship. *Religions* 12:650. 10.3390/rel12080650

[B6] Burgstahler S. (2009). *Universal design of instruction (UDI): Definition, principles, guidelines, and examples. DO-IT.* Available online at: https://www.washington.edu/doit/sites/default/files/atoms/files/UD_Instruction_06_15_20.pdf (accessed March 15, 2022)

[B7] Cihak D. F. Moore E. J. Wright R. E. McMahon D. D. Gibbons M. M. Smith C. (2016). Evaluating augmented reality to complete a chain task for elementary students with autism. *J. Spec. Educ. Technol.* 31 99–108. 10.1177/0162643416651724

[B8] Ekinci E. Acar F. E. (2019). Primary school teachers’ opinions on professional development (Professional Development Model Proposal). *J. Educ. Train. Stud.* 7 111–122. 10.11114/jets.v7i4.4039

[B9] Hakiman H. Bambang S. Watson W. (2021). Religious instruction for students with autism in an inclusive primary school. *Int. J. Learn. Teach. Educ. Res.* 20 139–158. 10.26803/ijlter.20.12.9

[B10] Hanafi Y. Murtadho N. Ikhsan M. A. (2020). Reinforcing public university student’s worship education by developing and implementing mobile-learning management system in the ADDIE Instructional Design Model. *Int. J. Interact. Mob. Technol.* 14 215–241. 10.3991/ijim.v14i02.11380

[B11] Hanum L. (2014). Pembelajaran PAI bagi anak berkebutuhan khusus. *Jurnal Pendidikan Agama Islam.* 11 217–236. 10.14421/jpai.2014.112-05

[B12] Katz J. (2013). The Three-Block model of universal design for learning (UDL): Engaging students in inclusive education. *Can. J. Educ.* 36 153–194.

[B13] King-Sears M. E. (2009). Universal design for learning: Technology and pedagogy. *Learn. Disabil. Q.* 32 199–201. 10.2307/27740372

[B14] Koo J. Y. M. Khairuddin K. F. (2021). “Sokongan pembelajaran dan halangan pelajar berkeperluan khas di institusi pengajian tinggi,” in *International Conference of Future Education and Advances (ICOFEA)* (Bandar Baru Bangi), 410–419.

[B15] Kpobi L. Swartz L. (2019). Ghanaian traditional and faith healers’ explanatory models of intellectual disability. *J. Appl. Res. Intellect. Disabil.* 32 43–50. 10.1111/jar.12500 29993171

[B16] Larco A. Carrillo J. Chicaiza N. Yanez C. Luján-Mora S. (2021). Moving beyond limitations: Designing the helpdys app for children with dyslexia in rural areas. *Sustainability* 13:7081. 10.3390/su13137081

[B17] Lee H.-J. Han C. Kim H.-J. Herner-Patnode L. (2021). Teaching multiplication to students with mathematical learning disabilities (MLD): Analysis of preservice teachers’ lesson design. *Sustainability* 13:11813. 10.3390/su132111813

[B18] Majoko T. (2019). Teacher key competencies for inclusive education: Tapping pragmatic realities of Zimbabwean special needs education teachers. *Sage Open* 9:2158244018823455. 10.1177/2158244018823455

[B19] Mateos-Sanchez M. Melo A. C. Blanco L. S. García A. M. F. (2022). Chatbot, as educational and inclusive tool for people with intellectual disabilities. *Sustain.* 14:1520. 10.3390/su14031520

[B20] Md Yusoff Y. Awang A. (2019). Kepentingan nilai-nilai Islam dalam pembangunan diri orang kelainan upaya (OKU) kategori bermasalah pembelajaran. *Jurnal Usuluddin* 47 105–118. 10.22452/usuluddin.sp2019no1.5

[B21] Ministry of Education Malaysia (2014). *Malaysia education blueprint: Annual report 2014.* Available online at: https://www.moe.gov.my/muat-turun/penerbitan-dan-jurnal/pppm-2013-2025-pendidikan-prasekolah-hingga-lepas-menengah/3925-annual-report-2014 (accessed March 15, 2022).

[B22] Mohd Khalil N. D. Shaberi M. Izhar D. P. N. Mat Rashid A. Mohd Rapini A. (2022). Analisis isu-isu berkaitan hak dan permasalahan orang kurang upaya di Malaysia: Satu sorotan literatur: Analysis on issues related to rights and difficulties of people with disabilities in Malaysia: A literature review. *J. Muwafaqat.* 5 33–51.

[B23] Mohd Jamil M. R. Siraj S. Mat Noh N. Sapar A. A. (2017). *Pengenalan Asas Kaedah Fuzzy Delphi dalam penyelidikan rekabentuk dan pembangunan.* Bangi: Minda Intelek Agensi.

[B24] Mohd Jamil M. R. Mat Noh N. (2021). *Kepelbagaian metodologi dalam penyelidikan rekabentuk dan pembangunan.* Bangi: Qaisar Prestige Resourses.

[B25] Murray T. J. Pipino L. L. Gigch J. P. (1985). A pilot study of fuzzy set modification of Delphi. *Hum. Syst. Manag.* 5 76–80. 10.3233/HSM-1985-5111

[B26] Mustapha R. Darussalam G. (2018). *Aplikasi kaedah fuzzy delphi dalam penyelidikan sains sosial.* Kuala Lumpur: University Malaya.

[B27] Onwumere D. D. Cruz Y. M. Harris L. I. Malfucci K. A. Seidman S. Boone C. (2021). The impact of an independence curriculum on self-determination and function in middle school autistic students. *J. Occup. Ther. Sch. Early Interv.* 14 103–117. 10.1080/19411243.2020.1799904

[B28] Ozcan D. Merdan F. (2020). The effectiveness of video modelling for teaching daily life skills to children with autism spectrum disorder. *Int. J. Learn. Teach.* 12 42–54. 10.18844/ijlt.v12i1.4560

[B29] Parr J. Stevens T. (2019). Challenges of equity and discrimination in the education of gifted children. *Qual. Educ.* 1 1–12. 10.1007/978-3-319-69902-8_21-1

[B30] Poston D. J. Turnbull A. P. (2004). Role of spirituality and religion in family quality of life for families of children with learning disabilities. *Educ. Train. Autism Dev. Disabil.* 39 95–108.

[B31] Schaap-Jonker H. Sizoo B. Van Schothorst-Van Roekel J. Corveleyn J. (2013). Autism spectrum disorders and the image of god as a core aspect of religiousness. *Int. J. Psychol. Relig.* 23 145–160. 10.1080/10508619.2012.688005

[B32] Story M. F. Mueller J. L. Mace R. L. (1998). *The Universal design file: Designing for people of all ages and abilities.* Raleigh, NC: NC State University, the Center for Universal Design.

[B33] Training and Development Agency for Schools (2009). *For secondary PGCE tutors and trainees: Including students with SEN and/or disabilities in secondary religious education. Training toolkit.* Available online at: http://dera.ioe.ac.uk/13807/1/religiouseducationre.pdf (accessed March 15, 2022).

[B34] Tang C. W. Wu C. T. (2010). Obtaining a picture of undergraduate education quality: A voice from inside the university, Springer. *High. Educ.* 60 269–286. 10.1007/s10734-009-9299-5

[B35] Vygotsky L. S. (1978). *Mind in society: The development of higher psychological processes.* Cambridge: Harvard University Press.

[B36] Wilson K. P. Landa R. J. (2019). Barriers to educator implementation of a classroom-based intervention for preschoolers with autism spectrum disorder. *Front. Educ.* 4:27. 10.3389/feduc.2019.00027

[B37] Wright J. C. Knight V. F. Barton E. E. (2020). A review of video modeling to teach STEM to students with autism and intellectual disability. *Res. Autism Spectr. Disord.* 70:101476. 10.1016/j.rasd.2019.101476

[B38] Zadeh L.A. (1965). Fuzzy sets. *Inf. Control.* 8 338–353. 10.1016/S0019-9958(65)90241-X

