# Supplementary material for: Designing the content of religious education learning in creating sustainability among children with learning disabilities: A fuzzy delphi analysis
Source: Front Psychol. 2022 Nov 22;13:1036806. doi: 10.3389/fpsyg.2022.1036806 (PMC9722727; doi:10.3389/fpsyg.2022.1036806)
Supplement: Supplementary file 1 [file Data_Sheet_1.PDF]

NO.			

UNIVERSITI SAINS ISLAM MALAYSIA

UNIVERSITI KEBANGSAAN MALAYSIA

KOLEJ UNIVERSITI ISLAM ANTARABANGSA SELANGOR

QUESTIONNAIRE
THE DEVELOPMENT OF THE CONTENT LEARNING RELIGIOUS EDUCATION TO CHILDREN WITH LEARNING DISABILITIES

This research consist of:

Section A: Demography

Section B: Development of the content of Religious Education to children with learning disabilities

We request cooperation from Prof. Dr./Prof. Associate Dr./Dr./Sir/Madam to provide accurate and authentic information related to personal background and information in the questionnaire provided.

The cooperation from Prof. Dr./Prof. Associate Dr./Dr./Mr./Ms. We really appreciate it. All information stated is confidential and will not be disseminated to any party.

Thank you.

SECTION A :

DEMOGRAFY

Please fill in the form

1.	Gender	Male [ ]<br>Female [ ]
2.	Level of Education	Degree [ ]<br>Master [ ]<br>PhD [ ]<br><br>Others : …………………………………………..
3.	Work	
4.	Institution	
5.	Experience of involvement with people with disabilities	Less 5 years [ ]<br>6 to10 years [ ]<br>11 to 15 years [ ]<br>16 years onwards [ ]

SECTION B :

DEVELOPMENT CONTENT OF RELIGIOUS EDUCATION AMONG CHILDREN WITH DISABILITIES

Fill the form base on seven scale provided

Scale						
Strongly disagree	Strongly disagree	Strongly disagree	Strongly disagree	Strongly disagree	Strongly disagree	Strongly disagree
**1**	**2**	**3**	**4**	**5**	**6**	**7**

**A. Rights to learn for children with disabilities**								
**NO**	**SUBJECT**	**SCALE**						
		**1**	**2**	**3**	**4**	**5**	**6**	**7**
A1	The obligation of the disabled to study religion is the same as the typical human beings							
A2	Being disabled is not a barrier for children with disabilities to learn the religion							
A3	Children with disabilities can learn the religion according to appropriate methods and techniques							
**B. Access to support to learn religion for children with disabilities**								
B1	Islamic Administrative Institution (Religious Department)							
B2	Mosque Institution							
B3	Non-Governmental Organisations							
B4	Preacher							
B5	Activist							
B6	Neighbourhood							
B7	Volunteers							
B8	Parents							
B9	Family							
B10	Education Institution							
**C. Universal design learning for children with disabilities**								
C1	The infrastructure provided to learn the religion is in accordance with the needs of the disabled.							
C2	The teaching materials used in the learning of religion are in accordance with the needs of the disabled.							
C3	The learning method of the religion is suitable for the needs of the disabled.							
C4	Religion learning curriculum is suitable for the needs of the disabled							

C5	The form of support to learn the religion is in accordance with the needs of the disabled.							
C6	The communication used to learn the religion is in line with the needs of the disabled.							
C7	The assessment conducted is in accordance with the strengths of the disabled.							
C8	The infrastructure provided can be used by all categories of disabled people.							
C9	Teachers can adapt the curriculum according to the needs of the disabled.							
**C. Learning style for children with disabilities**								
D1	Children with disabilities can learn religion visually according to their ability.							
D2	Children with disabilities can learn religion visually according to their ability.							
D3	Children with disabilities can learn religion by reading and writing depending on their ability.							
D4	Children with disabilities can engage in kinesthetic learning of the religion according to their ability.							
D5	Children with disabilities can learn religion by combining more than one learning style depending to their ability.							
**D.** Attitude learning for children with disabilities								
E1	Ensure the person, clothing, and place is in a neat and clean condition.							
E2	Selection of appropriate time.							
E3	Covering the aurat while studying religion.							
E4	It is fun to study religion.							
E5	Respect and glorify the mushaf.							
E6	Praying to avoid accidents.							
E7	Praying before learning.							
E8	Read the scriptures with civility.							
E9	Read the scriptures.							
E10	Trying to read and memorise scriptures.							
E11	Respect teachers who teach religion.							
